# Dr. George Seymour

**Published:** 1909-12

**Authors:** 


### OBITUARY

Dr. George Seymour, of Utica, died November 8, 1909, after a brief illness, aged 70 years. Dr. Seymour graduated from the medical college of the University of the city of New York, in 1865, and after serving as a surgeon in the Civil War, practised medicine in several cities of this state before settling in Utica.

